# *C. elegans* VANG-1 Modulates Life Span via Insulin/IGF-1-Like Signaling

**DOI:** 10.1371/journal.pone.0032183

**Published:** 2012-02-16

**Authors:** Sebastian J. Honnen, Christian Büchter, Verena Schröder, Michael Hoffmann, Yuji Kohara, Andreas Kampkötter, Olaf Bossinger

**Affiliations:** 1 Institute of Molecular and Cellular Anatomy, Medical School, RWTH Aachen University, Aachen, Germany; 2 Institute of Toxicology, Heinrich-Heine-University Düsseldorf, Düsseldorf, Germany; 3 Department of General Pediatrics, University Children's Hospital, Heinrich-Heine-University Düsseldorf, Düsseldorf, Germany; 4 Genome Biology Laboratory, National Institute of Genetics, Mishima, Japan; 5 Research and Development, Bayer Animal Health GmbH, Leverkusen, Germany; Brown University, United States of America

## Abstract

The planar cell polarity (PCP) pathway is highly conserved from *Drosophila* to humans and a PCP-like pathway has recently been described in the nematode *Caenorhabditis elegans*. The developmental function of this pathway is to coordinate the orientation of cells or structures within the plane of an epithelium or to organize cell-cell intercalation required for correct morphogenesis. Here, we describe a novel role of VANG-1, the only *C. elegans* ortholog of the conserved PCP component Strabismus/Van Gogh. We show that two alleles of *vang-1* and depletion of the protein by RNAi cause an increase of mean life span up to 40%. Consistent with the longevity phenotype *vang-1* animals also show enhanced resistance to thermal- and oxidative stress and decreased lipofuscin accumulation. In addition, *vang-1* mutants show defects like reduced brood size, decreased ovulation rate and prolonged reproductive span, which are also related to gerontogenes. The germline, but not the intestine or neurons, seems to be the primary site of *vang-1* function. Life span extension in *vang-1* mutants depends on the insulin/IGF-1-like receptor DAF-2 and DAF-16/FoxO transcription factor. RNAi against the phase II detoxification transcription factor SKN-1/Nrf2 also reduced *vang-1* life span that might be explained by gradual inhibition of insulin/IGF-1-like signaling in *vang-1*. This is the first time that a key player of the PCP pathway is shown to be involved in the insulin/IGF-1-like signaling dependent modulation of life span in *C. elegans*.

## Introduction

Wnt/planar cell polarity (PCP) is one of three identified Wnt signaling pathways, along with Wnt/ß–Catenin and Wnt/Calcium [1]. These signaling pathways are abundant in various developmental processes across the animal kingdom [2]–[6]. PCP is extensively studied in the *Drosophila* wing, or in the organization of ommatidia in the fly eye or hair follicles in mammalian skin. Six proteins were placed in the core PCP pathway, Frizzled (Fz), Dishevelled (Dsh), Diego (Dgo), Strabismus/Van Gogh (Stbm/Vang), Prickle (Pk) and Flamingo (Fmi). The signaling mediated by PCP core proteins during development contributes to the polarization alongside the epithelial anterior-posterior or proximo-distal axis and requires contrary clustering of PCP components at the respective cell cortex. As a consequence of PCP signaling, downstream effectors (e.g., the actin cytoskeleton) are polarized within individual cells that finally lead to well organized structures within the two-dimensional epithelial surface [7], [8]. PCP processes also shape three-dimensional tissues that do not exhibit obvious signs of planar polarity. Here, individual cells have to move in a specific direction or divide with a specific orientation, hence showing transient planar polarization (e.g., during mediolateral cell intercalation) required for morphogenesis of the neural tube in vertebrates [9], [10]. Novel components of PCP signaling have been identified in the recent years, and the number of crosslinks to other conserved pathways required for development is rising [11]–[13].

The *C. elegans* genome (http://www.wormbase.org) encodes a sole four-pass transmembrane protein, VANG-1 showing sequence similarities and conservation of overall domain architecture compared to the Strabismus/Van Gogh/Ltap proteins identified in *Drosophila*, *Xenopus* and mammals. Like in *Drosophila* and mammals, VANG-1 contains four hydrophobic transmembrane domains at its N-terminus and a consensus PDZ binding motif at its C-terminus [13]. VANG-1 was implicated in playing a minor role in B cell polarity in the *C. elegans* male tail [14]. However, it plays a major role in organ formation either by mediating correct intercalation of intestinal primordial cells during embryogenesis [13], [15] or by establishing ground polarity in vulval development [12]. Whereas PCP signaling required for morphogenesis is generally well understood, a more physiological role of this pathway with effects on metabolism has not been described so far.

*C. elegans* is a well-established model to study genes that contribute to the process of aging. The corresponding genes of “Age” mutants are referred to as gerontogenes [16]. These mutants share a specific catalog of defects, e.g., a minimum of 20% life span increase and resistance against certain stress factors like reactive oxygen species or heat. The *C. elegans* homolog of insulin receptor in mammals, *daf-2*, is one of the best described gerontogenes, and the signaling mediated by DAF-2 is well understood [17]–[19]. DAF-2 is capable to phosphorylate target substrates, e.g., AGE-1/AAP-1, a PI3 kinase that generates PI(3,4,5)P3 [20]–[22]. Via a phosphorylation cascade, downstream kinases PDK-1, AKT-1, AKT-2 and SGK-1 [23]–[25] are activated and in turn negatively regulate the forkhead transcription factor (FoxO), DAF-16 [26], [27]. Inhibition of DAF-2 signaling (e.g., by *daf-2* mutations or active insulin peptide signaling) leads to dephosphorylation, activation and accumulation of DAF-16 in the nucleus [28]. Consequently, transcription of DAF-16 targets that include genes involved in defence against stresses, DNA repair and metabolism lead to a higher resistance against stresses and significantly extension of life span [29]. Besides DAF-16, inhibiting insulin/IGF-1-like signaling also activates heat-shock transcription factor HSF-1 and phase II detoxification transcription factor SKN-1, a Nrf1/2/3 protein ortholog [30], [31].

In the present study, we identify VANG-1, the only *C. elegans* ortholog of the conserved PCP protein Strabismus/Van Gogh, as a gerontogene with a typical phenotype, including extended life- and reproductive span, multiple stress resistances, slow growth, reduced brood size and reduced lipofuscin accumulation. The *vang-1*–dependent life span extension and stress defences seem to be coordinated in the germline and mostly require *daf-16* and *skn-1* gene functions.

## Results and Discussion

### *vang-1* increases life span, stress resistance and reproductive span in *C. elegans*

**Figure 1:**
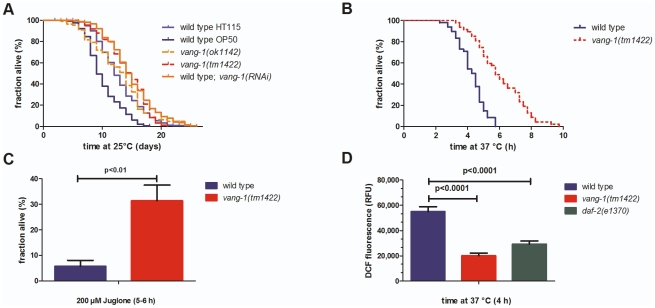
*vang-1* function interferes with life span extension and resistance against high temperature and reactive oxygen species in *C. elegans*. **(A)** ***vang-1*** **function interferes with life span extension in** ***C. elegans*** **.** *tm1422* (red), *ok1142* (green) and *vang-1(RNAi)* (orange) animals showed a significantly extended mean life span (**14.3±0.4 d**, n = 174*; **12.9±0.5 d**, n = 114*; and **14.9±0.2 d**, n = 576*, respectively) in comparison to controls: WT animals either grown on OP50 bacteria (light blue; **10.2±0.2 d**, n = 214*, p<0.0001**) or RNAi HT115 bacteria (blue; **12.8±0.1 d**, n = 936*, p<0.001**). **(B–D)** ***vang-1(tm1422)*** **increases resistance to thermal/oxidative stress in** ***C. elegans*** **.** (B) At 37°C, the mean survival time of *tm1422* (red, **6.2±0.3 h**, n = 48*) was significantly increased (p<0.01**) in comparison to WT (blue, **4.3±0.1 h**, n = 48*). (C) After 5–6 h under oxidative stress (induced by 200 µM juglone), a significantly larger fraction of *tm1422* animals survived (p<0,05***) (red, **34.4±6%**, n>100*) in comparison to WT (blue, **5.7±2%**, n>100*). (D) After 4 h at 37°C, *tm1422* animals (red, **20,020±2,148**, n = 48*) and *e1370* animals (green, **29,243±2,528**, n = 52*) showed a significantly lower DCF (2,7-dichlorofluorescein) fluorescence (p<0.001***) in comparison to WT (blue, **54,911±3,940**, n = 48*). (*three or more independent trials, **Mantel-Cox log rank test, ***unpaired t-test; animals grown on OP50 bacteria, if not stated otherwise; results are shown as mean**±**SEM).

**Figure 2:**
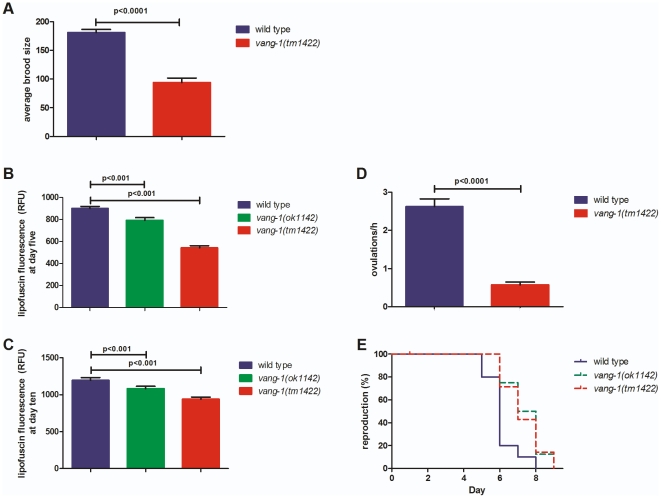
*vang-1* shows reproduction- and aging-related defects. **(A)** ***vang-1(tm1422)*** **populations have a reduced brood size.** The average brood size at 25°C in *vang-1(tm1422)* (red, **111±41** progeny; n = 28*) is significantly reduced (p<0.0001**) in comparison to WT (blue, **194±50** progeny; n = 56*). Results are shown as mean±standard deviation. **(B–C)** ***ok1142*** **and** ***tm1422*** **show decreased lipofuscin accumulation five and ten days after hatching.** (B) Five days after hatching, *ok1142* (green, **RFU = 792.35±25**, n = 31, p<0.001**) and *tm1422* (red, **RFU = 543.1±18**, n = 37, p<0.001**) accumulate significantly less lipofuscin in comparison to WT (blue, **RFU = 900.4±17.27**, n = 45). (C) Ten days after hatching, *ok1142* (green, **RFU = 1083±32**, n = 33, p<0.05**) and *tm1422* (red, **RFU = 940.9±27**, n = 29, p<0.01**) still accumulate significantly less lipofuscin in comparison to WT (blue, **RFU = 1196±37**, n = 27). Results are shown as mean±SEM of relative fluorescence units (RFU: OD_individual_−OD_background_/mm^2^). **(D) In** ***tm1422*** **the ovulation rate is reduced in comparison to WT.** *tm1422* has an ovulation rate of **0.7±0.1** (n = 25***) and the WT shows **2.3±0.7** (n = 15***) what is significantly more (p<0.05**). Ovulations were counted per gonad arm per hour at 20°C for synchronous WT and mutant populations. **(E)** ***vang-1*** **populations have a prolonged reproductive span.** The reproductive span in *ok1142* (green, **6.6** d; n = 20***) and *tm1422* (red, **6.9** d; n = 20***) is significantly prolonged (p<0.05^##^) in comparison to WT (blue, **5.7** d; n = 20***). (*three independent trials, **unpaired t-test, ***two independent trials; animals grown on OP50 bacteria, ^##^Mantel-Cox log rank test).

The *C. elegans* genome (http://www.wormbase.org) contains a sole four-pass transmembrane protein with homology to the Strabismus/Van Gogh/Ltap proteins identified in *Drosophila*, *Xenopus* and mammals [32]–[34]. During analysis of *vang-1(tm1422)*, in which 188 amino acids of the N-terminus are missing (including three of the four transmembrane domains; see supporting information S1) [13], we noticed several defects (Figs. 1, 2) with regard to the postembryonic phenotype, e.g., slow growth (data not shown) and reduced fecundity (Fig. 2A) that are also associated with loss of function phenotypes of certain aging genes in *C. elegans* [35]. Life span assays in *vang-1(tm1422)* at 25°C (Fig. 1A), 20°C and 18°C (Table 1) detected a significant increase in mean life span of up to 40% compared to wild type (WT) animals. Furthermore, we tested life span of another *vang-1* deletion mutation, *ok1142*, lacking 162 amino acids of the C-terminus (including a predicted phosphorylation site; see supporting information S1) [13] and of animals depleted of VANG-1 by RNAi (Fig. 1A). Again, we noticed a significant extension of *C. elegans* mean life span up to 27% and 20% in comparison to WT controls either kept on standard OP50 or RNAi HT115 bacteria with the empty “feeding”-vector. In addition, the *tm1422* phenotype was not enhanced by RNAi against *vang-1* C-terminus (Table 1). With regard to longevity we assume *tm1422* to be a null mutation whereas *ok1142* seems to be a hypomorphic allele and RNAi does not generate the complete loss-of-function phenotype, as reported for other genes [36]. Hence, we used *tm1422* in all further experiments.

**Table 1:** Summary of life spans.

	Background	Conditions	LS +/− SEM	N	Significance
1	WT	OP50	10.2+/−0.2	214	
2	*tm1422*	OP50	14.3+/−0.4	174	*(1)
3	*ok1142*	OP50	12.9+/−0.5	114	*(1)
4	WT	18°C/OP50	19.6+/−0.8	61	
5	*tm1422*	18°C/OP50	27.1+/−0.9	43	*(4)
6	WT	20°C/HT115	21.4+/−0.4	70	
7	*tm1422*	20°C/HT115	25.6+/−0.4	71	*(25)
8	WT	20°C/*daf-16(RNAi)*	19.5+/−0.5	75	*(25)
9	*tm1422*	20°C/*daf-16(RNAi)*	22.5+/−0.5	71	*(26) 0.72(25)
10	WT	HT115	12.8+/−0.1	936	*(1)
11	*tm1422*	HT115	15.8+/−0.2	480	*(10)
12	WT	*vang-1(RNAi)*	14.9+/−0.2	576	*(10)
13	*tm1422*	*vang-1(RNAi)*	14.9+/−0.4	242	0.37(11)
14	WT	*skn-1(RNAi)*	13.0+/−0.2	133	0.76(10)
15	*tm1422*	*skn-1(RNAi)*	13.2+/−0.5	138	*(11)
16	WT	*daf-16(RNAi)*	11.9+/−0.2	142	*(10)/0.27(17)
17	*tm1422*	*daf-16(RNAi)*	12+/−0.3	247	*(11)
18	WT	*daf-2(RNAi)*	25.8+/−1.1	126	*(10)/0.62(19)
19	*tm1422*	*daf-2(RNAi)*	25.6+/−1.3	135	*(11)
20	OLB11	HT115	14+/−0.3	250	*(10)
21	OLB11	*daf-2(RNAi)*	22.6+/−0.7	85	*(20)
22	OLB11	*vang-1(RNAi)*	14.4+/−0.3	195	0.24(20)
23	NL2098*(rrf-1)*	HT115	12.9+/−0.3	309	
24	NL2098*(rrf-1)*	*vang-1(RNAi)*	14.6+/−0.3	274	*(23)
25	TU3401	20°C/HT115	17.0+/−0.3	380	
26	TU3401	20°C/*vang-1(RNAi)*	17.7+/−0.3	284	0.86(25)
27	TU3311	20°C/HT115	19.5+/−0.5	212	
28	TU3311	20°C/*vang-1(RNAi)*	21.1+/−0.4	280	*(27)

Life spans (**LS**±SEM, standard error of the mean, at 25°C, if not stated otherwise) under different experimental conditions in WT, two different alleles of *vang-1* (*tm1422* and *ok1142*), the intestine-specific RNAi strain OLB11 [60], [61], germline-specific RNAi strain NL2098 [62] and the neuron-enhanced and neuron-specific strains TU3311 and TU3401 [64]. OP50 [75] and RNAi HT115 [77], [78] indicate standard and RNAi *E. coli* strains, respectively. Comparison of significant results are indicated by *(p<0.01; Mantel-Cox log rank test) with corresponding experiments in parentheses (the p-value is stated, if not significant). All the life span assays were repeated at least three times. Data shown is a sum of all experiments.

Next, we tested increased resistance of *tm1422* against various stressors, a typical feature of gerontogenes. First, we measured thermoresistance in semi-automated and manual assays using SYTOX® Green nucleic acid stain under lethal temperature conditions of 37°C. Both assays revealed increased thermoresistance of *tm1422* of about 40% (Fig. 1B and not shown), which is in the similar range as extension of mean life span. Second, we tested the resistance of *tm1422* against reactive oxygen species (ROS) and determined intracellular ROS accumulation in living worms. In a stress assay with juglone (from *Juglans niger*) as a redox cycler, we found the fraction of *tm1422* animals that survived the induced ROS stress conditions was about four fold higher than WT (Fig. 1C). Furthermore, a 60% decrease in ROS accumulation, which is in the similar range of *daf-2* mutant population (Fig. 1D), was found in *tm1422* worms in comparison to WT using a fluorescence well-plate reader to measure DCF fluorescence (see “methods” for details). According to the “free radical theory of aging” [37], ROS are a crucial factor for aging, and the intracellular amount of ROS can be correlated to stress. Organisms developed inducible detoxification systems like catalases, peroxidases and superoxide dismutases to reduce ROS levels [38]. The competence to keep intracellular ROS levels low is considered to be one possibility for the extension of life span [39], [40]. Thus, the diminished amounts of ROS in *tm1422* may explain the increased survival rate at lethal thermal stress conditions. Recent findings suggest that the relationship between ROS and the aging process is more complex than what was originally thought. The generation of ROS cannot be longer seen as the initial trigger of the aging process [41]. Nevertheless, in case of *tm1422* population reduced ROS generation indicates a lower stress level that finally may account for the extension of life span.

In order to estimate the biological age of *vang-1* mutants, we measured the amount of lipofuscin, a product of oxidative damage and autophagy. In *C. elegans*, lipofuscin is detectable as autofluorescent granules in the intestine and its accumulation is a well-established marker to judge the biological age of *C. elegans* [42], [43]. In comparison to WT, *ok1142* and *tm1422* animals showed a significantly decreased accumulation of lipofuscin after five (Fig. 2B) and even after ten days (Fig. 2C).

In addition to the longevity phenotype, we observed a significantly reduced brood size in *tm1422* animals (Fig. 2A), a decreased ovulation rate (Fig. 2D) and a dramatically prolonged reproductive span (Fig. 2E). Normally, the reproductive system of *C. elegans* ages significantly during the first week of adulthood [44], reflected by germline degeneration and a decline in oocyte quality [42], [45]. Individual *tm1422* mothers continue to produce viable progeny as they age (Fig. 2D). This phenotype also points to *vang-1* being a typical gerontogene. Some of the known mutations (e.g., *daf-4* or *daf-7*) that extend *C. elegans'* reproductive period also regulate longevity, suggesting that there is a link between reproductive span and life span [46].

Taken together these results suggest that loss of the planar cell polarity ortholog VANG-1 causes robust temperature independent extension of life span, increases stress resistance and extends reproductive period in *C. elegans*.

### Life span modulation by VANG-1 depends on the insulin/IGF-1-like signaling pathway

**Figure 3:**
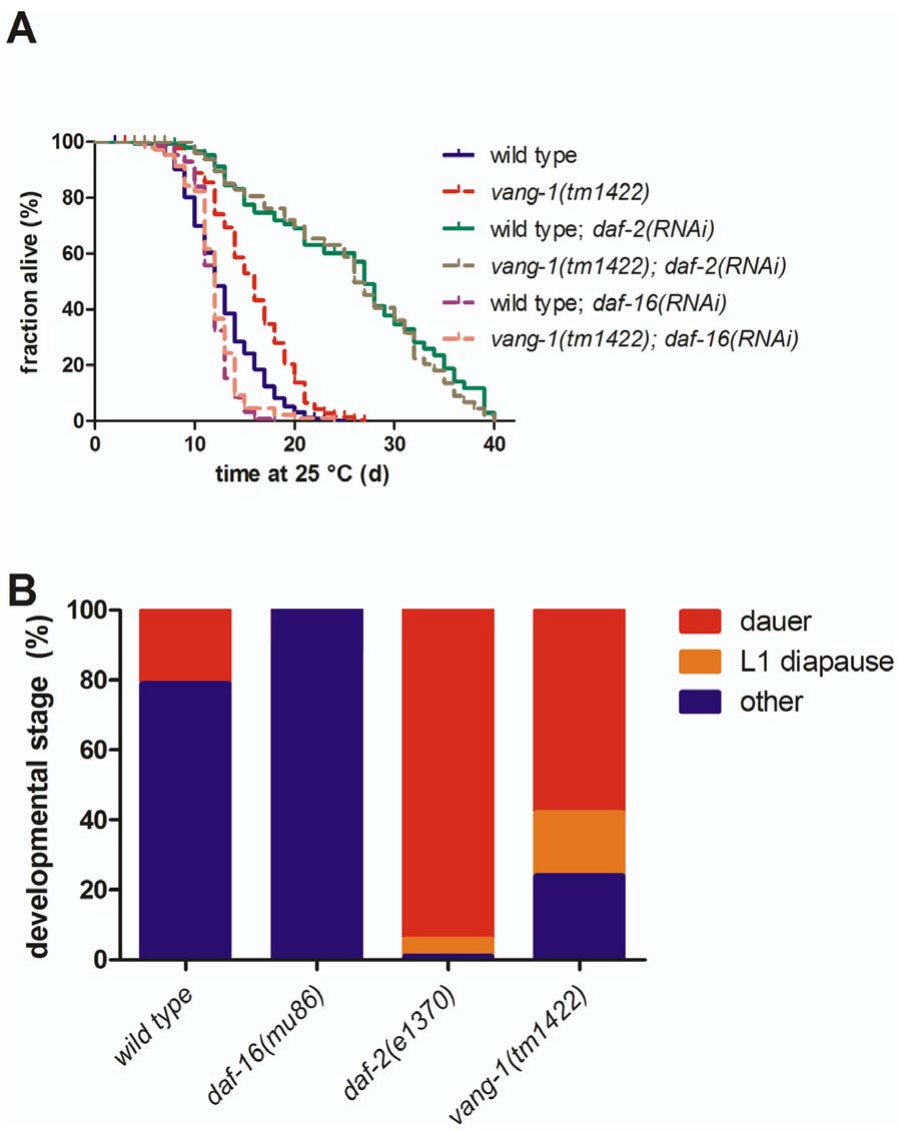
*vang-1(tm1422)* life span modulation depends on Insulin/IGF-1-like signaling and leads to higher DAF-16 activity. **(A)** ***vang-1(tm1422)*** **induced life span extension interferes with RNAi against** ***daf-2*** **and** ***daf-16*** **.** Depletion of DAF-2 by RNAi in *tm1422* (brown spotted line) and WT (green solid line) causes an increase of mean life span to **25.6±1.3 d** (n = 135*) and **25.8±1.1 d** (n = 126*), respectively (p<0.62**), which is in agreement with published results for *daf-2* mutants [23]. In contrast, depletion of DAF-16 by RNAi in *tm1422* (rose spotted line) and WT (purple solid line) causes a decrease of mean life span to **12±0.3 d** (n = 247*) and **11.9±0.2 d** (n = 142*), respectively (p<0.96**). Life spans of WT (blue solid line) and *tm1422* (red spotted line) fed with RNAi HT115 bacteria carrying the empty “feeding”-vector are **12.8±0.1 d** (n = 936*) and **15.8±0.2 d** (n = 480*), respectively (p<0.001**). **(B)** ***vang-1(tm1422)*** **populations are dauer constitutive.** Synchronous populations were scored after 60 h at 27°C (OP50 bacteria) for dauers and L1 in diapause. All farther grown and adult animals were pooled as “other”. WT animals developed **21%**, **0%** and **79%** dauers, L1 diapause and “other”, respectively (n = 390*). *daf-2(e1370)* animals showed **94.7%**, **4.6%** and **0.7%** dauers, L1 diapause and “other”, respectively (n = 281*). *daf-16(mu86)* animals developed **100%** “other” (n = 111*). *tm1422* showed **57.6%**, **18%** and **24.4%** dauers, L1 diapause and “other”, respectively (n = 205*, p<0.05^§^). (*three or more independent trials, **Mantel-Cox log rank test, animals grown on OP50 bacteria, if not stated otherwise, ^§^Data analyzed by Chi-square test).

The main regulator of longevity and stress resistance in *C. elegans* is insulin/IGF-1-like signaling with its effector DAF-16. This FoxO transcription factor is translocated into the nucleus where it activates gene expression for distinct processes, e.g., resistance against different stressors and longevity when insulin/IGF-1-like signaling is inhibited [47], [48]. To gain further insight into the pathway operating in *tm1422*, we disrupted FoxO/DAF-16 transcription factor by RNAi in *tm1422* and WT worms and compared the mean life span (Fig. 3A and Table 1). As expected [49], mean life span in WT animals depleted of DAF-16 slightly decreased in comparison to the control. Surprisingly, *daf-16(RNAi)* in *tm1422* eliminated *vang-1* induced life span extension at 20°C and 25°C (Table 1), suggesting that *daf-16* is epistatic to *vang-1*.

The activation of the DAF-16 transcription factor can be easily observed by a functional DAF-16::GFP fusion [50]. After *vang-1(RNAi)* at room temperature and 27°C we observed 16% and 57% DAF-16 translocation into the nucleus, respectively (Fig. S1), suggesting that complete nuclear localization of DAF-16 is not a prerequisite for increased life span and stress resistance. This phenomenon has also been observed in case of *age-1* at 20°C, which is well known for modulating life span in a DAF-16 dependent manner [48], [50].

To further validate our *daf-16(RNAi)* life span result, we investigated other parameters of high DAF-16 activity (e.g., developmental arrest). In *C. elegans*, the activity of DAF-16 is sufficient and necessary for L1 diapause and dauer formation [25], [51]. Hatching L1 larvae stay in diapause, a developmental arrested state with reduced metabolism, until they start feeding. Dauer formation is an alternative third larval stage (beside the normal L3 larval stage) that is introduced under harsh environmental conditions, high temperature, low food or overcrowding [52].

We performed our dauer assay in comparison to WT, *daf-2(e1370)* and *daf-16(mu86)* at 27°C [53]. Consistent with the literature, we found that *daf-2(e1370)*, encoding the sole insulin receptor homologue in *C. elegans* [20], is dauer constitutive (∼99% arrest), while *daf-16(mu86)* is dauer defective (0% arrest; Fig. 3B) [51], [54]. *tm1422* animals showed four times more developmental arrest compared to WT (Fig. 3B), which is inhibited by RNAi against *daf-16* (*tm1422*: 7.8% dauer, 92.2% “other”, n = 64; WT: 1.2% dauer, 98.8% “other”, n = 160;). While 21% of WT animals developed into dauers, 58% and 18% of *tm1422* animals arrested as dauers and in L1 diapause, respectively (Fig. 3B). A noteworthy difference concerning the dauer constitutive phenotypes of *daf-2* and *tm1422* is the percentage of L1 diapause arrests, which is also induced by DAF-16 [51] and suggests higher activity of DAF-16 in *tm1422* during early development.

We further investigated the role of the receptor tyrosine kinase DAF-2 [20], which acts upstream of FoxO/DAF-16 transcription factor to modulate life span and stress resistance in the conserved insulin/IGF-1-like signaling pathway [55]. Inhibition or loss of DAF-2 function leads to induction of alternate dauer formation (see above) early in life and life span extension of up to 100% late in life both depending on DAF-16 function [29]. RNAi against *daf-2* in WT and *tm1422* worms resulted in nearly identical survival curves with no significant difference in mean life span (Fig. 3A and Table 1), indicating that *vang-1* may function in the insulin/IGF-1-like signaling pathway, rather than in parallel pathways, e.g., through regulation of DAF-16 by *kri-1* and lipophilic-hormone signaling [56], [57].

We also tested the longevity promoting factor SKN-1/Nrf2, which orchestrates the phase II detoxification response including defense against oxidative stress [58]. RNAi against *skn-1* did reduce *tm1422* life span significantly about 17% (Table 1). Inhibition of insulin/IGF-1-like signaling in *tm1422* may explain this result. Like DAF-16, SKN-1 is also repressed by DAF-2 downstream kinases, AKT-1/2 and SGK-1 and possibly acts as a key player in a positive feedback loop to extend life span [58], [59].

To further specify how *vang-1* functions in the extension of life span, we performed specific knock downs of *vang-1* first in the intestine [60], [61], second in the germline [62] and third, because of its expression in ventral cord neurons [12], [63], in strains showing enhanced neuronal RNAi [64].

**Figure 4:**
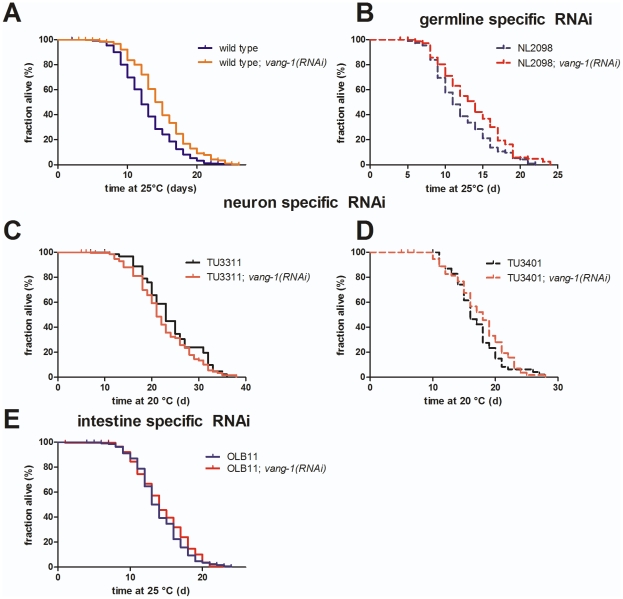
Tissue specific RNAi against *vang-1*. **(A)** ***vang-1*** **function interferes with life span extension in** ***C. elegans*** **.** *vang-1(RNAi)* (orange) animals showed a significantly extended mean life span (**14.9±0.2 d**, n = 576*) in comparison to control: RNAi HT115 bacteria (blue; **12.8±0.1 d**, n = 936*, p<0.001**). **(B) Germline-specific RNAi against** ***vang-1*** **effects** ***C. elegans*** **life span.** After *vang-1(RNAi)* in germline-specific RNAi strain NL2098 a significant increase (13%) of mean life span (**14.6±0.3 d**, red spotted line, n = 274*) in comparison to the control (NL2098 kept on RNAi HT115 bacteria carrying the empty “feeding”-vector) can be observed (**12.9±0.3 d**, blue spotted line, n = 309*, p<0.01**). **(C–D) Neuron-specific RNAi against** ***vang-1*** **does not effect** ***C. elegans*** **life span.** After depletion of VANG-1 in the enhanced-neuronal RNAi strain TU3311 *([unc-119p::YFP+unc-119p::sid-1])*, the mean life span is **21.1±0.4 d** (orange solid line, n = 280*) compared to **19.5±0.5 d** (green solid line, n = 92*, p = 0.02**) in the control (TU3311 kept on RNAi HT115 bacteria carrying the empty “feeding”-vector). The same is true in the neuron-specific RNAi strain TU3401 *(sid-1(pk3321) V; [pCFJ90(myo-2p::mCherry)+unc-119p::sid-1])*, which only has SID-1 in neurons. Depletion of VANG-1 in this strain leads to a mean life span of **17.7±0.3 d** (red spotted line, n = 284*) and **17±0.3 d** (blue spotted line, n = 380*, no significant difference**) in the control (TU3401 kept on RNAi HT115 bacteria carrying the empty “feeding”-vector). **(E) Intestine-specific RNAi against** ***vang-1*** **does not effect** ***C. elegans*** **life span.** After depletion of VANG-1 in the intestine-specific RNAi strain OLB11 {*rde-1(ne219);[pOLB11(elt-2p::rde-1)+pRF4(rol-6(su1006))]*}, the mean life span is **14.4±0.3 d** (red solid line, n = 195*) compared to **14.0±0.3 d** (blue solid line, n = 250*, no significant difference**) in the control (OLB11 kept on RNAi HT115 bacteria carrying the empty “feeding”-vector). (*three or more independent trials, **Mantel-Cox log rank test).

The intestine is highly exposed to environmental toxins and pathogens and it has been speculated to be the major site of stress response [65]. To further support this hypothesis, we depleted DAF-2 (as a control) by RNAi only in the intestine and found a 60% extension of life span (Table 1). In contrast, *vang-1(RNAi)* in the intestine did not result in a significant extension of mean life span (Fig. 4A; Table 1), suggesting that the intestine is not where VANG-1 is acting to modulate life span.

In *C. elegans* and mice, VANG-1 and Vangl2^Lp^ have been connected with correct uterine epithelium development in the reproductive tract [12], [66], but its function in meiotic maturation and ovulation is still ellusive. Both processes are regulated by intense signaling between the germline and the proximal gonadal sheath cells, specialized myo-epithelial cells that surround and form gap junctions with oocytes [67]–[70]. During ovulation, sheath cells contract rapidly, the distal constriction of the spermatheca dilates, and sheath cells pull the distal spermatheca over the mature oocyte [71]. The decreased fertility/brood size, ovulation rate, and the increased reproduction span of *tm1422* animals (Fig. 2 A,D–E) suggests VANG-1 being involved in the communication between germline and somatic gonad. To test if *vang-1* also acts in the germline to control life span by insulin/IGF-1-like signaling, we performed germline-specific RNAi [62]. *vang-1(RNAi)* in *rrf-1* led to a significant increase in life span (13.5%, Fig. 4B; Table 1), which is about two third of whole life span extension observed in *vang-1(RNAi)* animals (Fig. 1A; Table 1). In contrast, depletion of VANG-1 in the enhanced-neuronal RNAi strains TU3311 and TU3401 [64], has no effect on *C. elegans* life span extension (Fig. 4C; Table 1). As suggested by Calixto et al. [64] the neuronal expression of *sid-1* in TU3311 might serve as a sink for double-stranded RNA used by non-neuronal RNAi and thus could explain why *vang-1(RNAi)* in TU3311 leads not to the same life span extension as in WT. Additionally, *vang-1(tm1422)* individuals have an intact chemosensory apparatus and are “open” to the environment (personal comunication with N.J. Storm - it has been testet two times with up to 30 individuals per experiment for uptake of DiI [72]). Dye-fill defective (dyf-phenotype) mutants have previous been found long-lived [73]. Taken together, our findings of tissue-specific RNAi against *vang-1* in combination with in-situ hybridization data of *vang-1*, *daf-2* and *daf-16* (Fig. S2) implicate the germline to be the primary site of *vang-1* action concerning longevity in *C. elegans*. Components of the insulin/IGF-1-like signaling pathway have already been implicated to act in the germline, e.g., Michaelson et al. found that the effect of reducing *daf-2* signaling on larval germline proliferation is dependent on *daf-16* [74].

In summary, we have identified a link between the *C. elegans* planar cell polarity key player *vang-1* and insulin/IGF-1-dependent extension of life span. Mutations in *vang-1* show the typical phenotype of age-mutants, including longevity, slow growth, multiple stress resistances, reduced lipofuscin accumulation, and reduced brood size. The germline, but not the intestine or neurons seems to be the primary site of *vang-1* function, which may operate in the same pathway as *daf-2* and *daf-16* to extend life span of about 40% in *C. elegans*.

## Methods

### *C. elegans* strains and alleles

Maintenance and handling of *C. elegans* were carried out as described previously [75]. Bristol N2 was used as the WT strain. WT or mutant worms were synchronized as described previously [76].

#### Single mutants were as follows

TM1422: *vang-1(tm1422)X* (outcrossed ×3); RB1125: *vang-1(ok1142) X*; CB1370: *daf-2(e1370) III*; CF1038: *daf-16(mu86) I*; NL2098: *rrf-1(pk1417) I*.

#### Transgenic strains were as follows

OLB11: *rde-1(ne219);[pOLB11(elt-2p::rde-1)+pRF4(rol-6(su1006))]*; TU3311: *[unc-119p::YFP+unc-119p::sid-1]*; TU3401: *sid-1(pk3321) V; [pCFJ90(myo-2p::mCherry)+unc-119p::sid-1]*; TJ356: integrated DAF-16::GFP roller strain [50] (for further details see: https://cgcdb.msi.umn.edu/strain.php?id=13306).

### RNA-mediated interference (RNAi)

RNAi by “feeding” was performed essentially as described by others [77]. In brief, after amplification of a single colony overnight (37°C, LB_amp tet_ medium), HT115(DE3) bacteria (RNase III-deficient *E. coli* strain, carrying IPTG-inducible T7-polymerase) [77], [78] were diluted to an OD_600_ of 0.9, and after addition of IPTG (1 mM) seeded on NGM_amp tet_ plates (containing 1 mM IPTG). Bacteria were further incubated overnight at room temperature (∼22°C) to allow the expression of double-stranded RNA. HT115(DE3) bacteria harboring the empty KS+ based vector L4440 (containing two T7 promoters flanking a polylinker) were used as a control for RNAi “feeding” experiments. RNAi clones against *vang-1* and *daf-16* were obtained from the Ahringer RNAi “feeding”-library (Geneservice Limited, Cambridge, UK) while *daf-2* “feeding”-clone was kindly provided by Dr. Andrew Dillin [79] (see supporting information S2 for sequencing results of RNAi “feeding”-clones).

### Life span assay

Life span was determined at 25°C, if not stated otherwise. Because *vang-1(tm1422)* shows a delayed egg laying phenotype, synchronization was performed as follows: embryos were randomly collected from cut-off worms, transferred and grown on plates (three plates per trial) either seeded with OP50 [75] or HT115(DE3) [77], [78] bacteria harboring the empty L4440 “feeding”-vector or L4440 with a fragment of the gene of interest [77]. Worms were transferred to fresh plates every day during time of reproduction but at least every third day. Individuals were considered as dead when stopped moving and not responded to gentle touches. When dying upon “rupture”/“bag of worms” phenotypes or disappearance occurred, the animal was censored on that day. The resulting data sets were analyzed using Kaplan-Meier survival test and weighted log-rank tests [80].

### Determination of progeny

*C. elegans* populations were synchronized and hatched on NGM Agar plates at 25°C. On day three, single worms were transferred as L4 larvae to 35 mm NGM-plates with NGM agar. Adult worms were transferred to fresh plates and then their progenies were counted each day. The experiment was stopped when production of progeny ceased.

### Dauer assay

The assay was performed as described elsewhere [53]. In brief, some gravid adults were put on individual tagged 60 mm NGM-plates where they laid eggs for 4–6 h at 20°C before they were removed again. Plates were shifted to the assay temperature of 27°C. After 60 h the stages were scored for L1 diapause and dauers. Farther grown worms (individuals larger than L2 larvae but not predauer/dauer stages) were pooled as “other”.

### Reproductive span of self-fertile animals (modified after [46])

Ten hermaphrodites per trial were individually transferred to fresh 35 mm NGM-plates seeded with OP50 daily. No production of progeny for 48 h marked reproductive cessation. Individuals were censored if they died or matricide occurred. All trials were conducted at 20°C with age synchronized populations. Unpaired t-test was used to test null hypothesis.

### Determination of ovulation rate (modified after [71])

Documentation of ovulation rates were performed using a Zeiss Axioplan 2 microscope. Age-synchronized worms with more than six oocytes in-utero were transferred to small agarose pads on a microscope slide and coated with a cover slip. The number of ovulated oocytes per animal was counted for 3 h and slides were kept in a moisture chamber at room temperature.

### DAF-16::GFP translocation

Synchronized populations of TJ356 (DAF-16::GFP) [50] worms were kept for 72 h at 25°C on NGM plates seeded with RNAi HT115 bacteria either carrying the empty “feeding”-vector or a fragment of *vang-1* cDNA. 15 Individuals per trial were transferred to small agarose pads (3%) on a microscope slide, anesthetized with levamisole (1%), coated with a cover slip, illuminated with UV light under an Axiolab fluorescence microscope (Zeiss, Göttingen, Germany) and dedicated to three categories concerning DAF-16::GFP translocation: “cytoplasmatic” (uniform distribution of DAF-16::GFP), “intermediate” (clearly distinguishable DAF-16::GFP in some nuclei), and “nuclear” (DAF-16::GFP in nearly all nuclei with low background fluorescence).

### Lipofuscin accumulation

WT *C. elegans* were synchronized, hatched on NGM Agar plates at 20°C and transferred to fresh plates every second day. At day five and day ten, individuals were placed on microscope slides, anaesthetized with 20 mM sodium azide in M9 buffer [76] and coated with a cover slip. Epifluorescence (excitation, 365 nm; emission, 420 nm) images were taken with Image ProPlus software (Version 4.5, MediaCybernetics, Silver Spring, MD, USA) using a CoolSnap CF Digital Monochrome Camera (Intas, Göttingen, Germany) mounted on an Axiolab fluorescence microscope (Zeiss, Göttingen, Germany) and using a 100× oil immersion objective. The fluorescence intensity of individual worms was determined densitometrically as relative fluorescence units (RFU: OD_individual_−OD_background_/mm^2^).

### Assessment of resistance to thermal/oxidative stress and determination of intracellular ROS accumulation in *C. elegans*

The resistance of WT and mutant animals to thermal stress was assessed by a semi-automated assay according to [81] with some modifications described in [82]. After synchronization [76] both strains were cultured on NGM plates with OP50 bacteria [75] for five days at 20°C. Worms were then washed in PBST (PBS/0.1% Tween 20) and individually transferred with 1 µl PBST to the wells of a 384-well microtiter plate (Greiner Bio-One, Frickenhausen, Germany, #788096) containing 9 µl PBST with 1×10^7^ OP50 bacteria/ml [82]. Immediately after transfer 10 µl of 2 µM SYTOX® Green nucleic acid stain (Molecular Probes Inc., Leiden, Netherlands) in PBS was added to the wells and the plate was sealed using BackSeal-96/384 Black (Perkin Elmer, Wellesley, USA, #6005189) to avoid evaporation. SYTOX® Green can only enter cells with compromised plasma membranes and exerts a bright fluorescence in the DNA-bound state. Therefore, the fluorescence intensity is an indicator for cellular damage and hence for the viability of worms [81]. For the application of thermal stress the fluorescence reader (Wallace Victor^2^ 1420 multilabel counter, Perkin Elmer, Wellesley, USA) was preheated to 37°C. The measurement of each well through the transparent bottom of the microtiter plate (excitation, 485 nm; emission, 535 nm) was carried out for a minimum of 13 h with intervals of 15 min and a 0.2 s integration time. Fluorescence curves for every single well were obtained and individual cut off values were determined by multiplying the background fluorescence (average of the first four measurement readings) by a factor of three [81]–[83]. The time point when fluorescence exceeded the cut off value was defined as the point of death of the corresponding worm and the survival curves as well as the mean life spans were assessed from these individual times of death.

To compare resistance to oxidative stress WT and mutant animals synchronized [76] and L4 larvae were incubated for approximate 5 h at 20°C in liquid NGM containing 200 µM juglone, a redox cycler that generates intracellular oxidative stress [84]. Worms were then allowed to regenerate on NGM plates with OP50 bacteria [75] for about 20 h at 20°C before viability was determined by touch provoked movement [85].

For the determination of the intracellular amount of ROS synchronized WT and mutant larvae were cultured as described above and individually transferred with 1 µl PBST to the wells of a 384-well microtiter plate containing 7 µl PBS [86]. After the complete transfer of the individual worms 2 µl 250 µM 2,7-dichlorodihydrofluorescein diacetate (H_2_DCF-DA; Molecular Probes Inc., Leiden, Netherlands) in PBS (final concentration, 50 µM) was added to the wells and the plate was sealed (see above). After entering cells H_2_DCF-DA is intracellular converted to membrane-impermeable, non-fluorescent H_2_DCF, which then can be oxidized by ROS to yield fluorescent DCF and thus is a marker for the individual amount of intracellular ROS in a single worm [82], [83], [86]. The fluorescence of each well is then measured through the transparent bottom in a fluorescence reader (see above) every 15 min for a minimum of 13 h at 37°C (1.0 s integration time; excitation, 485 nm; emission, 535 nm).

## Supporting Information

Figure S1 **DAF-16::GFP translocation into the nucleus.** In TJ356 (DAF-16::GFP) worms [50], RNAi against *vang-1* at room temperature (RT) led to **12%** and **4%** intermediate and nuclear localization of DAF-16::GFP, respectively (n = 49*). In contrast, TJ356 control animals fed with RNAi HT115 bacteria, carrying the empty “feeding”-vector, showed **100%** cytoplasmic localization of DAF-16::GFP (n = 70*). Under heat stress condition (27°C), *vang-1(RNAi)* causes **45%** and **12%** intermediate and nuclear localization of DAF-16::GFP, respectively (n = 42*). In comparison, TJ356 control animals showed **42%** intermediate- and **3%** nuclear localization of DAF-16::GFP (n = 95*). *(p<0.05 by two-way ANOVA with Bonferroni's post hoc test; three or more independent trials).
(TIF)

Figure S2 **Expression patterns in** ***C. elegans*** **adults of** ***daf-2*** **(A),** ***daf-16*** **(B) and** ***vang-1*** **(C) genes.** All images represent *in situ* hybridization to endogenous transcripts (enriched in the gonad, arrows) and are taken from the Nematode Expression Data Base (http://nematode.lab.nig.ac.jp/db2/index.php). Scale bars: 60 µm.
(TIF)

Supporting Information S1 **Sequences of VANG-1, VANG-1^tm1422^ and VANG-1^ok1142^ proteins.** Missing amino acids in *tm1422* and *ok1142* are shown in red and blue, respectively. Additional amino acids in *ok1142* are shown in yellow. For further details concerning VANG-1 see [13].
(DOCX)

Supporting Information S2 **Sequences of RNAi “feeding”-clones.**
(DOCX)
