# Supplementary figures and images for: C. elegans VANG-1 Modulates Life Span via Insulin/IGF-1-Like Signaling
Source: PLoS One. 2012 Feb 16;7(2):e32183. doi: 10.1371/journal.pone.0032183 (PMC3281126; doi:10.1371/journal.pone.0032183)

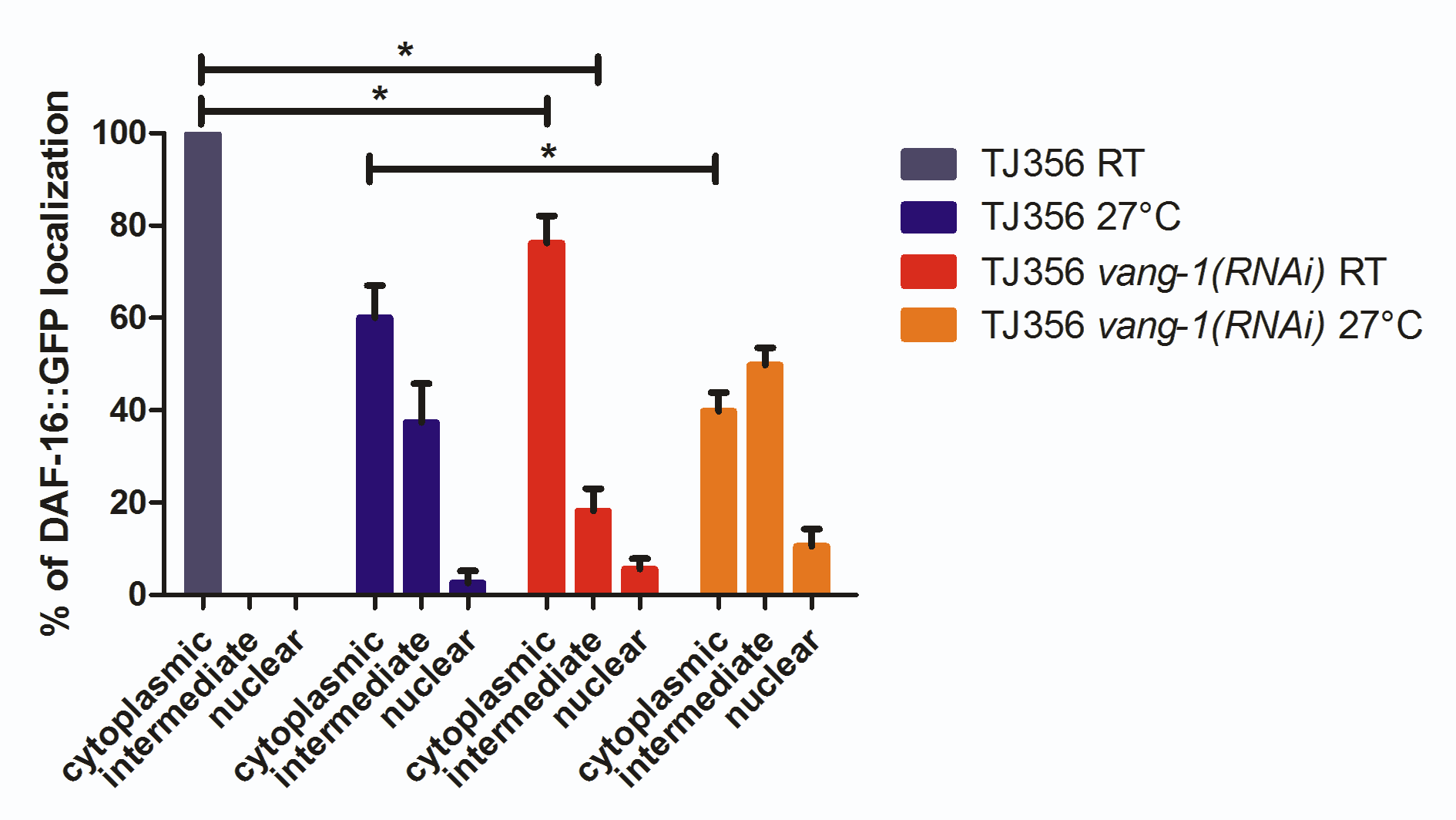

Supplement: Figure S1 — DAF-16::GFP translocation into the nucleus. In TJ356 (DAF-16::GFP) worms [50], RNAi against vang-1 at room temperature (RT) led to 12% and 4% intermediate and nuclear localization of DAF-16::GFP, respectively (n = 49*). In contrast, TJ356 control animals fed with RNAi HT115 bacteria, carrying the empty “feeding”-vector, showed 100% cytoplasmic localization of DAF-16::GFP (n = 70*). Under heat stress condition (27°C), vang-1(RNAi) causes 45% and 12% intermediate and nuclear localization of DAF-16::GFP, respectively (n = 42*). In comparison, TJ356 control animals showed 42% intermediate- and 3% nuclear localization of DAF-16::GFP (n = 95*). *(p<0.05 by two-way ANOVA with Bonferroni's post hoc test; three or more independent trials). (TIF) [file pone.0032183.s001.tif]

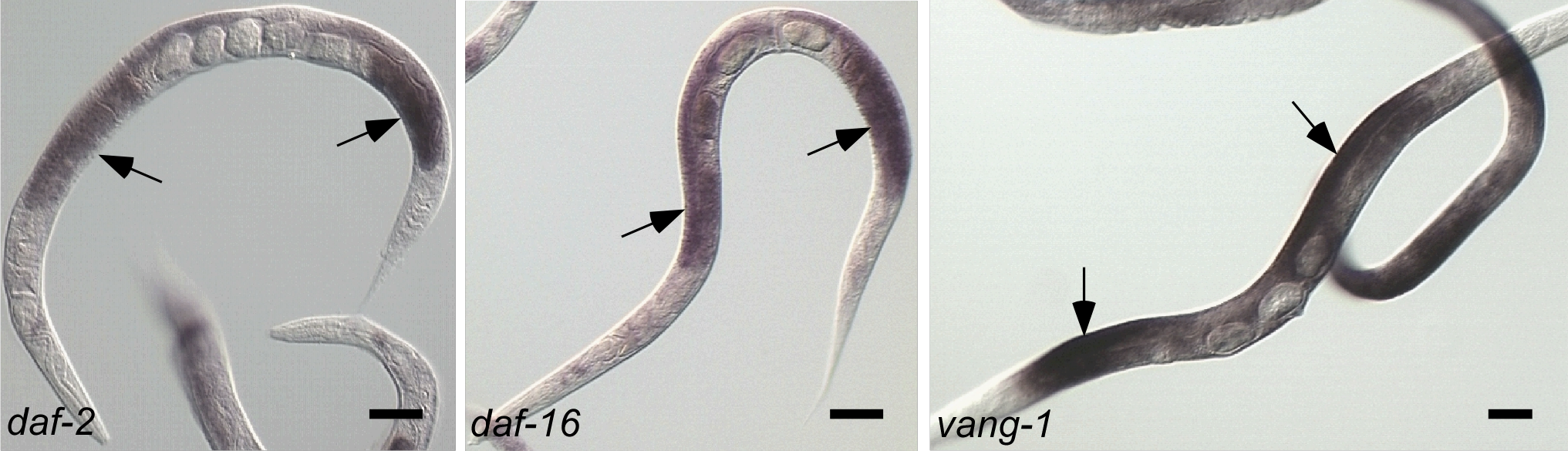

Supplement: Figure S2 — Expression patterns in C. elegans adults of daf-2 (A), daf-16 (B) and vang-1 (C) genes. All images represent in situ hybridization to endogenous transcripts (enriched in the gonad, arrows) and are taken from the Nematode Expression Data Base (http://nematode.lab.nig.ac.jp/db2/index.php). Scale bars: 60 µm. (TIF) [file pone.0032183.s002.tif]
