# Supplementary material for: C. elegans VANG-1 Modulates Life Span via Insulin/IGF-1-Like Signaling
Source: PLoS One. 2012 Feb 16;7(2):e32183. doi: 10.1371/journal.pone.0032183 (PMC3281126; doi:10.1371/journal.pone.0032183)
Supplement: Supporting Information S1 — Sequences of VANG-1, VANG-1tm1422 and VANG-1ok1142 proteins. Missing amino acids in tm1422 and ok1142 are shown in red and blue, respectively. Additional amino acids in ok1142 are shown in yellow. For further details concerning VANG-1 see [13]. (DOCX) [file pone.0032183.s003.docx]

MSYQDNRKLPKDTRSCVGGFRYEGHKKQLRPRYAQSEIGEPFIPRFSAIASEGQKIAPPNEDWADNTTVLTGMTTDSFTMEEKVIYTPPIGRVIGRRCSRFVWLLASSLLCIISVVSAPIMCSLPIIAPRFGFSMPAIQCDVDCEGLLFLMAIKTIFLVIAIGVLYWRKAMADMPRLYFVRAALTFLVMFILFAFWLFYIVRIMFERYDNYKYIVSYSTSLLDALLWTHYLSVVLLELRRLRAQFIVTIVRDPDGEMHTLNIGAGSIQEAATEILRFYTTRFSSFNIHLDNARQTAVAKQSGMQGGTAGFKMYNIEQFGGQETVSEVNTRALMEAAARRRIGGYAEVMQEELDFEKRLKKRKYRLIAAAEDAFSHVQNTAESGTNQKPGINNQMDSLTAAQNVFTWIVRPLTKYLKTTRLQSRHPSGEVTRHIERCLTLKLSHRTFLQRFFSDRIPQREIVGESKWSVICDEAVSSGVQHGTYLVLKSHNPNTLTKNITNKIRKLDIDCGVQLVCTISSIPFFNLTEQAKPGNEKFSLKISNESAV
