# Supplementary material for: C. elegans VANG-1 Modulates Life Span via Insulin/IGF-1-Like Signaling
Source: PLoS One. 2012 Feb 16;7(2):e32183. doi: 10.1371/journal.pone.0032183 (PMC3281126; doi:10.1371/journal.pone.0032183)
Supplement: Supporting Information S2 — Sequences of RNAi “feeding”-clones. (DOCX) [file pone.0032183.s004.docx]

***vang-1* / (B0410.2)**

AGCCGACGACCGAGCGCAGCGAGTCAGTGAGCGAGGAAGCAACCTGGCTTATCGAAATTAATACGACTCACTATAGGGAGACCGGCAGATCTGATATCATCGATGAATTCGAGCTCCACCGCGGTGGCGGCCGCTCTAGAACTAGTGGATCCACCGGTTCCATGGCTAGCCACGTGACGCGTGGATCCCCCGGGCTGCAGGAATTCGATATCAAGCTTATCGATACCGTCGACCTCGAGATGGTGCAAACAAGTTGAACTCCGCAGTCGATATCTGGGTTGTGCGATTTGAGCACTAGATACGTTCCGTGTTGAACTCCAGAGCTCACCGCCTCGTCGCAAATTACCGACCATTTCGATTCCCCGACAATTTCCCGTTGGGGAATACGGTCACTGAAGAAGCGTTGGAGAAATGTTCGATTCTTTCAATGTGCCTCGTAACTTCTCCTGAAGGATGTCTTGACTGAAGACGAGTAGTTTTTAAATATTTGGTGAGGGCCGGACAATCCATGTGAATACGTTTTGAGCAGCGGTCAGTGAGTCCATCTGGTTGTTAATTCCAGGCTTTTGGTTCGTTCCGGACTCGGCAGTGTTCTGAACATGGGAGAATGCGTCTTCGGCGGCGGCAATTAGGCGATACTTTCTCTTCTTTAACCGTTTTTCAAAATCAAGTTCTTCCTGCATCACTTCAGCATATCCACCAATCCTTCTTCTCGCCGCGGCTTCCATCAAAGCCCGCGTGTTGACTTCACTAACAGTTTCCTGCCCTCCAAATTGCTCGATATTGTACATCTTGAAGCCTGCGGTGCCTCCTTGCATTCCAGACTGCTTTGCAACCGCCGTCTGTCTGGCGTTGTCAAGGTGAATGTTGAACGAGGAGAATCGAGTTGTGTAGAAACGAAGAATCTCGGTGGCAGCTTCTTGAATTGAGCCAGCGCCATGTTCAA

***daf-16* / (R13H8.1)**

CGCGTATACGACTCACTATAGGGCGAATTGGGTACCGGGCCCCCCCTCGAGGTCGACGGTATCGATAAGCTTGATTTTGGATTTCGAAGAAGTGGATTCTGAGCACACGATCCACGGACACTGTTCAACTCGTGGTATGATGGTGGTGGAGCAATTGGTTCCGTCTGAAAATTCTATATATTACTCAGTTTTTTCACTTAAAAAAAAAGAAAATTCTAATTTTCGGTGTGAGAAAATATAAAATGATTTGCATGTTACTAATATTTTAGGGGACCTTGAAACTAAATTCAGAATTAAAAATATTGAATTTAAGTAAAATTCAAATTTTTTAATTGTCTAAAAAGGAGCTCACTTGTACTAACCACAAGAAAATTTTCAAATTTTCCTGAAAATTGCAAATTCTCATTTCTCACCTTAATCGGCTTCGACTCCTGCTTAATCTGAACTCCACCAATATGAGTAGTTGCATCGATACGCATTTGATCAGTTCTATCAACAATATCACTTGGAATTGCTGGAACCGATTCGCCAACCCATGATGGGAATTCTAGATCATCATAGATATCACTTCCAATAGCTGGAGAAACACGAGACGACGATCCAGGAATCGAGAGGTTCGATTGAGTTCGGGGACTGAAAAAATTTGGAAATTTGGAAAATCCAAAAAATAAAAATAAAACTAAATTACTAACCGGAAAGATGATGGAACGTTATCAAATGCTCCTTGCATTGAATCATCATCATACAAATCGTGAGAAATCGTTTGAATCGATCCGGCAATCGAATTTCCATTAAGTGTCGAGTGAAGGGAGCCCATCAATGCTCTCTCCTTTATCCTCTTCTTGGCTCCGCGGCGAGATTTTTCGAGTTGAGCCTGAAATTTTAAGCGTATTTATAGTGAGGTGAAATTACCAAATATCATAGTAAAAATTTTCGAAAAATTCTAGGAAAACTTTTAATTTGAGTCGAAAAGCTGAGAAATCCATTTTAGCTCATTTAGCACTATAAAATTATTTCACTATCTCTACCT

***daf-2* / (Y55D5A.5)**

ACGACCGAGCGCAGCGAGTCAGTGAGCGAGGAAGCGGAAGAGCGCCCAATACGCAAACCGCCTCTCCCCGCGCGTTGGCCGATTCATTAATGCAGCTGGCACGACAGGTTTCCCGACTGGAAAGCGGGCAGTGAGCGCAACGCAATTAATGTGAGTTAGCTCACTCATTAGGCACCCCAGGCTTTACACTTTATGCTTCCGGCTCGTATGTTGTGTGGAATTGTGAGCGGATAACAATTTCACACAGGAAACAGCTATGACCATGATTACGCCAAGCGCGCAATTAACCCTCACTAAAGGGAACAAAAGCTGGAGCTCGCGCGCGTAATACGACTCATATAGGGGCGAAGCACTAGTGGATCCCCCGGGCTGCAGGAATTCGCTCGCGCGGTCCAATATCCATAAAAATGTCCGGGGTCGGTTCACCGTTCGACGTCTCGTAGTACTGTTTGAAGACACTCTGCCACGAATCGACACACGCACTTCGATCCTCTTCGATCGTCATGTTCTCATCGATTCGTGGGACTTCTTTGAAGAAGAGCTCGTAGCCGAGAAACTTTCGCTGATCTATATCGGTAATGTTGAATGAGGGCCAACTAAAGAAGACCGAGTCCGCGTTGACCGCTGTGATGCTCACGTTGATTGCCATATCCTCACAGATTGCCTTCTCACCATTTGTCCCTTCTGATTGATCTATCGGATCGAGTGGTATATTTAACTTTGACATTAGCTGCTTGATATACTTGAAGCATAACATCTTGTTATTGGCAATTGACACAGTTCCACGATCAAGCGTCAAATCCGTCGTTGAATCGAATAGCTTTTTTTAAATTC

***skn-1* / (T19E7.2)**

CGCGTATACGACTCACTATAGGGCGAATTGGGTACCGGGCCCCCCCTCGAGGTCGACGGTATCGATAAGCTTGATTGGGTACCCACTTGCCCTATTTGAGCAAGTGCACTTTTACTAATAAGCTGAATGGGATCCCAAGAGACTTTCTAGACTTTCAGACTGAGCAACTTACGTAGTGCAATCACTAAACGAGTGTCTCTGTGAGTGATATGGATCGAACAACGATTGAGTAGCTGTTGCAGTCACTGTGACGCTTCCTGGGCTCGTTTGAGTCTGCTGTTGACGTCCTGAAGATCCAATGATGAGAGGAGACAGTGGAGTCTGACCAGTGGATTGATAGGAATGATCATAGTCAATCTGTCCATTTGATACAACTGCTTGAAGACTGTCGTTTTGCATTCCAATGTAGGCGTAGTTGGATGTTGGGAACACTGGAAATTTTTATAATTATAAGAAAACTGAAAGGTATGGGTTTCAAACTCTCTTATATCTTGAAATATTTTAAGCCGCTTCTAGGTTAACAACCCTCTGCCATTCCAAGATATGAGGGAGCCCTGAAAGGAGGAAATGTTGGAATGTCTAGATAGGATGAAAGAGAGAGAGAGAGAGAGAGAGAGATAGGAACCTCTGTCAAAAGTGCTCGAGACTCCTGAATAGGCGTATGGATGTTGGTGATGATGGCCGTGTTGATCCACCTGTTGTTGATTGAACATTTGAAGGTACATGTTCAGTATCTTCTTGGATTCTTCTTCTTGTTCGTCAAAAGAGTTGACAGCTGCTGGAGCGGTGTTGGTGGTGTTGGTGGGG
